# A cytoderm metabolic labeling TPAPy-Tre for real-time detection of vitality of *Mycobacterium tuberculosis* in sputum

**DOI:** 10.1128/spectrum.02457-24

**Published:** 2025-05-22

**Authors:** Mengru Yang, Guiqin Dai, Dan Li, Pengfei Zhao, Senlin Zhan, Hongjuan Qin, Hongzhou Lu, Mingbin Zheng, Peize Zhang

**Affiliations:** 1 School of Public Health, Shenzhen University Medical School, Shenzhen University 47890, Shenzhen, Guangdong, China; 2 Shenzhen Clinical Research Center for Tuberculosis, Shenzhen, China; 3 Institute of Hepatology, National Clinical Research Center for Infectious Diseases, Third People’s Hospital of Shenzhen, Shenzhen, Guangdong, China; 4 National Clinical Research Center for Infectious Diseases, Shenzhen Clinical Research Center for Tuberculosis, Shenzhen Third People’s Hospital, Shenzhen, Guangdong, China; 5 Department of Pulmonary Medicine and Tuberculosis, Third People’s Hospital of Shenzhen, Shenzhen, Guangdong, China; 6 Department of Infectious Diseases, National Clinical Research Center for Infectious Diseases, Third People’s Hospital of Shenzhen, Shenzhen, Guangdong, China; City of Hope Department of Pathology, Duarte, California, USA

**Keywords:** early bactericidal activity (EBA), tuberculosis (TB), TPAPy-Tre microscopy, fluorescence intensity, treatment response monitoring

## Abstract

Early bactericidal activity is a vital measure in developing new tuberculosis (TB) drugs. Traditional methods, including colony-forming units (CFUs) and time to positivity (TTP), have limitations. This study aimed to evaluate the efficacy of TPAPy-Tre fluorescence microscopy in monitoring early therapeutic responses compared with conventional culture methods in patients with pulmonary multidrug-resistant/rifampicin-resistant TB. In an open-label clinical trial at the Third People’s Hospital of Shenzhen, China, seven sputum smear-positive patients aged ≥18 years were enrolled. Sputum samples were consecutively analyzed using solid and liquid cultures and TPAPy-Tre microscopy. The study found that TPAPy-Tre fluorescence intensity significantly decreased from 271.5 (95% confidence interval [CI], 177.4–365.7) before treatment to 142.8 (95% CI, 104.7–180.9) after treatment (*P* < 0.05). TPAPy-Tre results strongly correlated with CFU (Spearman ρ = 0.60; 95% CI, 0.35–0.77; *P* < 0.001) and TTP (Spearman ρ = −0.33; 95% CI, −0.56 to −0.04; *P* < 0.05). Among selected participants, the median fluorescence intensity decreased from 51.5 (interquartile range [IQR], 39.0–63.6) to 13.2 (IQR, 7.8–20.0) after treatment (*P* < 0.001). TPAPy-Tre shows potential as a rapid, visual method for tracking bacterial vitality during TB treatment, offering immediate feedback on treatment response. These results support its use alongside conventional methods in clinical settings, though larger studies are needed for further validation.

**IMPORTANCE:**

Early bactericidal activity (EBA) is an important tool in clinical studies in the development of new tuberculosis drugs. Current traditional methods of efficacy monitoring present significant limitations. There is a need for novel and efficient tools to monitor treatment response in real-time when EBA is performing.

## INTRODUCTION

Tuberculosis (TB) remains one of the most significant infectious diseases worldwide, with an estimated 10 million new cases and 1.5 million deaths annually (1). The emergence of multidrug-resistant tuberculosis (MDR-TB) further complicates the global TB crisis, necessitating the development of new and more effective drugs (1–4).

Early bactericidal activity (EBA) is an important and valuable tool in clinical studies in the development of new TB drugs (5, 6). It is widely used to assess the antimycobacterial activity of new drugs and drug combinations in patients with pulmonary TB, to guide dosing, and to provide preliminary information about drug safety and tolerability in patients with active TB (5–10). Traditional methods for evaluating EBA, such as serial sputum colony counts, are labor-intensive and time-consuming (11). Time to detection of live *Mycobacterium tuberculosis* (Mtb) in a liquid culture system has been developed as an alternative to colony counting in the last decade (12). It is labor-friendly but requires sophisticated laboratory infrastructure (11). Therefore, there is a need for innovative and more efficient tools to monitor treatment response when EBA is performed. In the past several years, the development of novel diagnostic tools like the tuberculosis molecular bacterial load assay (TB-MBLA) and clustered regularly interspaced short palindromic repeats-mediated tuberculosis (CRISPR-TB) assay has shown promise in rapid TB bacteria detection and accurate quantification of viable Mtb (11, 13). While TB-MBLA generates informative quantitative results for treatment response, it is still largely manual and demands substantial hands-on time for RNA extraction (11). CRISPR-TB reflects material released from dying Mtb and apoptotic cells in real time, rather than accumulated material reflecting earlier Mtb burden (13).

Recently, a fluorescein-modified trehalose analog (FITC-Tre) enabled the rapid and accurate detection of Mtb in sputum samples from TB patients by participating in glycolipid biosynthesis, embedding into the live bacterial cell wall, allowing for real-time monitoring of bacterial load and treatment response (14–16). Trehalose monomycolates and trehalose dimycolates are the most abundant components of the mycomembrane of Mtb and are recognized as essential for maintaining Mtb cellular viability (16, 17). These glycolipids are anchored in the cytoderm through catalysis by the antigen-85 (Ag85) complex (18, 19). Utilizing this biological process, TPAPy-Tre, a trehalose derivative conjugated with fluorescein, was designed as an exogenous substrate to selectively aggregate onto the Mtb cytoderm, thereby reflecting bacterial viability (Fig. S1). TPAPy-Tre may offer a novel approach for imaging live bacteria in sputum samples, providing insights into the real-time change in the bacterial viability during anti-TB treatment.

In this study, we assess the efficacy of TPAPy-Tre in combination with conventional methods for monitoring EBA in the sputum of patients with MDR-TB undergoing new drug clinical trials. By juxtaposing this innovative approach with traditional methods, we aim to demonstrate the potential of TPAPy-Tre as a novel tool in TB treatment monitoring.

## MATERIALS AND METHODS

### Study design and participants

We conducted a prospective, open-label study in multidrug-resistant/rifampicin-resistant TB (MDR/RR-TB) patients from December 2023 to December 2025 at Third People’s Hospital of Shenzhen, China. Sputum smear-positive patients who were aged >18 years and met the criteria were enrolled in the EBA study. In this study, patients were randomly assigned to either the contezolid 800 mg twice daily group, contezolid 400 mg twice daily group, or linezolid 600 mg daily group in a 2:2:1 ratio for 14 days to verify early bactericidal activity. Linezolid is a proven robust therapeutic agent against tuberculosis. Contezolid has been demonstrated to have bactericidal activity against Mtb *in vitro* and *in vivo* and might be a novel drug for MDR/RR-TB. The trial was carried out following the Declaration of Helsinki and was registered at the Chinese Clinical Trial Registry (https://www.chictr.org.cn/) (ChiCTR2300074581).

To verify the diagnostic and treatment response monitoring performance of TPAPy-Tre for TB. Another study was embarked on in May 2023, with the clearance of the Ethics Committee of Third People’s Hospital of Shenzhen (no. IBS2023-043). These two studies were conducted prospectively in our hospital around the same time. We thus nested the TPAPy-Tre study within the EBA study.

### Sample processing

Sputum for EBA study was collected from 16:00 to 8:00 overnight from days –2, –1, 1, 2, 3, 4, 5, 6, 7, 8, 10, 12, and 14 of treatment. To measure numbers of colony-forming units (CFUs), samples were homogenized by magnetic stirring and combined with dithiothreitol (1:20 dilution; Sputasol, Oxoid, Cambridge, UK) to a maximum of 10 mL total volume, vortexed for 20 s, and left to digest at room temperature for 20 minutes. This mixture was plated in 10-fold dilutions onto 7H11S agar plates containing polymyxin B, amphotericin B, and trimethoprim. Numbers of CFUs were counted after positive culture, which were mostly reported 2–4 weeks after inoculation.

To measure time to positivity (TTP), homogenized sputum was decontaminated with NaOH-NALC (DZ0802; Leagene, Beijing, China), centrifuged, and resuspended, and 0.5 mL of the resulting 2 mL was used for incubation in duplicate in a standardized liquid culture system (BACTEC MGIT 960; Becton Dickinson, Franklin Lakes, USA). TTP was extracted from BACTEC software

### TPAPy-Tre assay-based sputum test

Sputum for the TPAPy-Tre study was collected from 16:00 to 8:00 overnight, consistent with the culture. Sputum samples were digested with NaOH-NALC (DZ0802; Leagene, Beijing, China) for 15–20 minutes to reduce the sample viscosity, eliminate the proteins and cells around Mtb, and kill other bacteria while keeping Mtb (with a tight and thick cytoderm structure) alive. Then the samples were filtered using a 40 µm filter (SCS402, Smtra, Hangzhou, China) and centrifuged at 4,000 rpm for 15 minutes and resuspended in phosphate buffered saline (PBS). Subsequently, the samples were incubated with TPAPy-Tre (40 µM) to label Mtb. For probe labeling, 100 µL of the sample was mixed with the probe solution and incubated at 37°C for 2 h. After labeling, the samples were centrifuged at 12,000 rpm for 5 minutes and resuspended in PBS. The labeled Mtb was observed using confocal laser scanning microscopy, and the fluorescence intensities of TPAPy-Tre anchored on Mtb were quantitatively analyzed by a microplate reader.

### TPAPy-Tre microscopy

TPAPy-Tre microscopy was performed on a Zeiss LSM980 confocal laser scanning microscopy using the 60×/1.4 oil objective and 10× ocular lens giving 600× magnification. Each membrane was evenly divided into eight sections, with a random selection of four sections examined under a 600× magnification. Upon identification of bacilli, digital images were captured using Carl Zeiss image software, followed by subsequent analysis of the images.

### Statistical analysis

We conducted normality tests on the acquired data and applied distinct descriptive statistics based on the data characteristics. For data that did not conform to a normal distribution, we utilized medians and interquartile ranges (IQRs) to describe the data. Conversely, for data that conform to a normal distribution, we used the mean with precise 95% confidence intervals (CIs) for our descriptions. We summarized participant characteristics using medians, IQRs, and proportions. The TPAPy-Tre-labeled fluorescence intensities before and after treatment were presented as mean, employing precise 95% CIs, assuming a normal distribution. We used the paired *t*-test to compare the TPAPy-Tre-labeled fluorescence intensities before and after tuberculosis treatment, as this involved the same individuals at two different time points. Comparisons of data with a normal distribution between two groups that were not paired were analyzed using unpaired *t*-tests. Otherwise, comparisons between two groups that did not meet the normal distribution and were not paired were analyzed using the nonparametric Mann–Whitney U-test. We analyzed data using Spearman’s ρ tests for bacterial load (time to positivity, CFU count, fluorescence intensity) due to the variables not conforming to a normal distribution. Trend testing was conducted on the results of consecutive tests. All tests were two-sided with an α of 0.05. Trend testing was performed using IBM SPSS 26, while all other analyses were conducted with GraphPad Prism (version 9.5.1). A *P*-value of less than 0.05 was considered statistically significant (***P < 0.001, **P < 0.01, *P < 0.05).

## RESULTS

A total of seven participants were enrolled and completed the EBA study in our trial. The median age of the seven participants was 47 years (IQR, 40–69), and all of them were male. The median erythrocyte sedimentation rate (ESR) was 79.5 mm/h (IQR, 42.3–91.8), and the median C-reactive protein (CRP) level was 22.5 mg/L (IQR, 4.6–82.6). The diagnosis of tuberculosis was confirmed in all participants by both mycobacteria growth indicator tube (MGIT) culture (six [100%] of six) and solid culture (seven [100%] of seven), with rifampicin resistance determined by Gene Xpert MTB/RIF (Cepheid, USA) (seven [100%] of seven). The majority of participants presented with at least two signs and symptoms based on the National Institutes of Health (NIH) criteria (four [57%] of seven), which include persistent cough, fever, night sweats, weight loss, and other respiratory symptoms (Table 1). The basic characteristics of all participants were presented in Table S1.

**TABLE 1:** Baseline characteristics and TPAPy-Tre-labeled fluorescence intensity in MDR/RR-TB patients^*c*^

	Number of participants with results	Overall (*n* = 7）
Age (years)	7	47 (40–69)
Sex		
Female	0	0
Male	7	7 (100%)
Clinical presentation		
ESR (mm/h)	6	79.5 (42.3–91.8)
CRP (mg/L)	7	22.5 (4.6–82.6)
Tuberculosis features		
NIH criteria signs and symptoms of TB >2^*a*^	7	4 (57%)
Positive IGRA	5	5 (100%)
Positive respiratory solid culture	7	7 (100%)
Positive respiratory MGIT culture	6	6 (100%)
Positive Xpert	7	7 (100%)
Number of patients positive for TPAPy-Tre	7	7 (100%)
Fluorescence intensity (a.u.) at earliest available visit and after TB treatment initiation (*n* = 9), mean (95% CIs)		
Before TB treatment	7	271.5 (177.4–365.7)
After TB treatment^*b*^	7	142.8 (104.7–180.9)
*P*-value	7	<0.05

^*a*^ Persistent cough, coughing up blood, chest pain, unintentional weight loss, fatigue or weakness, fever, night sweats, loss of appetite, and shortness of breath.

^*b*^ As compared with before tuberculosis treatment and after tuberculosis treatment initiation, as established by paired *t*-test.

^*c*^ Data are median (IQR) or *n* (%). IGRA, interferon-gamma release assay; Xpert, Xpert MTB/RIF; FL intensity, fluorescence intensity; a.u., arbitrary units.

**Fig 1:**
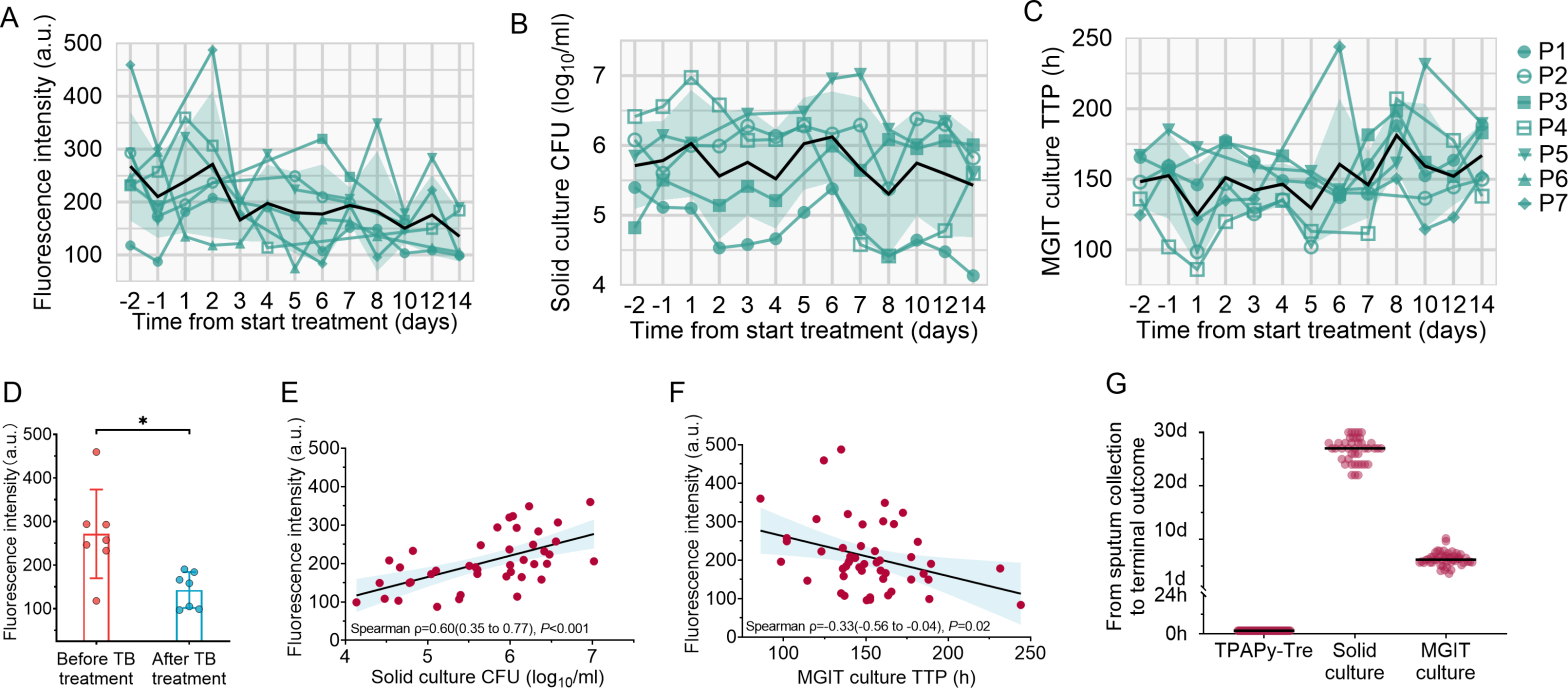
TPAPy-Tre evaluation in MDR/RR-TB patients within the initial 14 days of anti-TB therapy. (**A through C**) Quantitative results of fluorescence intensity, CFU, and TTP by time point. Lines connect data from individual participants. (**D**) TPAPy-Tre fluorescence intensity variation in seven MDR/RR-TB patients after 14-day treatment, with two-sided *P*-values from the paired *t*-test. Mean (95% CIs) presented for each group, *P < 0.05. (**E, F**) Quantitative information for fluorescence intensity of TPAPy-Tre compared with CFU or TTP. Solid lines are the linear regression line, and shaded areas are 95% CIs. (**G**) Time required to reach terminal outcomes from sputum collection using TPAPy-Tre, solid culture, and MGIT culture.

Of the 91 patient time points evaluated across seven patients at 13 time points, solid medium and MGIT culture results were available for 59 (65%) and 65 (71%), with missing data due to sample contamination. TPAPy-Tre results were available for 65 (71%) of the time points, with data missing due to some patients’ inability to produce sputum at certain time points. All participants had positive results in solid and MGIT culture, and TPAPy-Tre successfully identified all positive cases before TB treatment (Fig. S3). Measurements were conducted at distinct time points across a 14-day period for each of the seven patients, with TPAPy-Tre results available after a 2 h incubation. During the 14-day monitoring period, the fluorescence intensity of Mtb in sputum samples, labeled by TPAPy-Tre, showed a response to treatment, with a decline over time in all patients, *P* trend = 0.01 (Fig. 1A and Table 2). Concurrently, CFU counts and TTP mirrored the therapeutic response, exhibiting a reduction in bacterial load and an extension of TTP over the course of treatment, *P* trend = 0.045 for TTP, *P* trend = 0.437 for CFU (Fig. 1B and C and Table 2). In short, all the tests showed a response to treatment, with Mtb bacillary load tending to decrease immediately or to spike initially and then decrease after TB treatment, except the trend of CFU decrease was not significant (Fig. 1A through C and Table 2). Additionally, TPAPy-Tre sputum analysis in seven participants revealed a significant mean fluorescence intensity drop, from 271.5 (95% CI, 177.4–365.7) pre-treatment to 142.8 (95% CI, 104.7–180.9) post-treatment, showing about twofold fluorescence reduction (Fig. 1D and Table 1). When assessing the TPAPy-Tre and sputum bacillary load during the treatment period, the TPAPy-Tre-labeled fluorescence intensity correlated with CFU (Spearman ρ, 0.60; 95% CI, 0.35–0.77; *P* < 0.001) and TTP (Spearman ρ, −0.33; 95% CI, −0.56 to −0.04; *P* = 0.02), showing a proportional relationship with both culture methods (Fig. 1E and F). TPAPy-Tre provides results rapidly within 2 h post-sputum collection, whereas the median times for obtaining results are 27.0 days (IQR, 24.5–28.0) for solid culture and 6.2 days (IQR, 5.7–6.9) for MGIT culture (Fig. 1G).

**TABLE 2:** Associations between time from start of treatment and fluorescence intensity, CFU, and TTP among MDR/RR-TB patients within the initial 14 days of anti-TB therapy

	Fluorescence intensity	Solid culture CFU	MGIT culture TTP
	Cases	Cases	Cases
Time from start of treatment (days)			
Q1 (0 [<2])	24	18	20
Q2 (4 [2–5])	11	13	13
Q3 (7 [5–8])	15	14	16
Q4 (12 [8–14])	15	14	16
*P* trend	0.010	0.437	0.045

**Fig 2:**
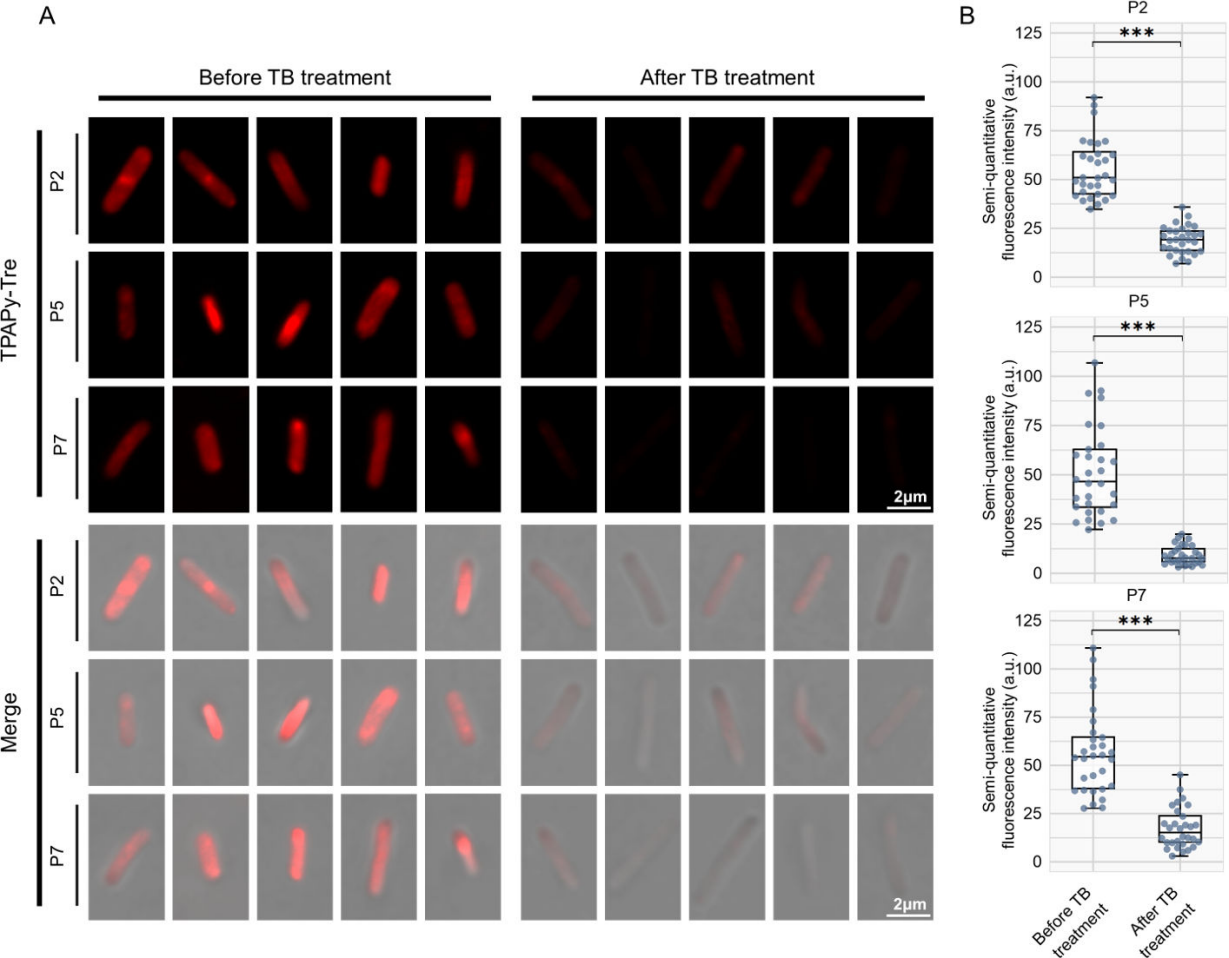
Confocal laser scanning microscopy analysis of real-time changes in bacterial sugar metabolism labeling in sputum with TPAPy-Tre. (**A**) TPAPy-Tre microscopy of Mtb. Images from several patients are shown, ordered by treatment phases. (**B**) Boxplots of bacterial fluorescence intensity for each participant at before and after treatment time points are presented, ***P < 0.001.

Fluorescence intensity, which reflects consistency with traditional culture methods macroscopically, prompts our further analysis of bacterial morphologies and fingerprint characteristics, with the expectation of discerning new patterns. Moreover, TPAPy-Tre microscopy revealed metabolic activity changes in individual bacilli, notably in fluorescence intensity (Fig. 2A). TPAPy-Tre microscopy measured bacilli images from participants P2, P5, and P7, showing a significant decrease in median fluorescence intensity from 51.5 (IQR, 39.0–63.6) before to 13.2 (IQR, 7.8–20.0) after TB treatment (*P* < 0.001). Individual post-treatment declines were consistent (Fig. 2B). The median cell length of bacilli from all three participants was 2.7 µm (IQR, 2.4–3.0) before TB treatment, showing a significant increase to 3.3 µm (IQR, 3.0–3.9) after TB treatment (Fig. S4). Most bacilli were 2–4 µm long curved rods, but bacilli of longer lengths up to approximately 5.6 µm were seen (Fig. S4). TPAPy-Tre microscopy has illustrated that metabolic activity changes at the single-bacterial level correspond with macroscopic changes in fluorescence intensity.

## DISCUSSION

In our study, we demonstrated that TPAPy-Tre is capable of monitoring changes in *Mycobacterium tuberculosis* metabolic activity in real-time. TPAPy-Tre may play as a viable alternative to traditional methods for evaluation of early bactericidal activity of new anti-TB drugs. Advancements in this novel and simple method might significantly enhance the efficiency of EBA studies, thereby accelerating the development and optimization of new anti-TB drugs.

The efficacy of anti-TB drugs is traditionally evaluated by monitoring the viable count of *Mycobacterium tuberculosis* in sputum samples (20, 21). When exposed to susceptible drugs, the activity of *Mycobacterium tuberculosis* declines exponentially (12). Traditional EBA studies involve continuous collection of sputum over 16 h, followed by solid and liquid culture, which were then evaluated by CFU counting and TTP, respectively (9). Both methods indirectly reflect the bactericidal action of the drug through the growth of *Mycobacterium tuberculosis* and have been shown to provide comparable evaluations of bactericidal activity (6, 12). Both methods were labor-intensive, resource-demanding, and time-consuming (11, 22). Furthermore, the issue of bacterial and fungal contamination during sample processing and culture poses a significant challenge, potentially compromising the accuracy and reliability of the results. Given these limitations, there is a pressing need to develop more streamlined and efficient methodologies for real-time monitoring of *Mycobacterium tuberculosis* activity.

Advances in molecular diagnostic techniques, such as the TB-MBLA and CRISPR-TB assay, have significantly improved the real-time evaluation of EBA of anti-tuberculosis drugs (1, 11, 13). TB-MBLA offers rapid results, high sensitivity and specificity, and minimizes contamination risk by quantifying viable *Mycobacterium tuberculosis* directly from sputum samples using quantitative real-time polymerase chain reaction (qRT-PCR) (11). Studies demonstrated that TB-MBLA can report the number of viable bacteria in 4 h (23). However, it requires specialized equipment, technical expertise, and stringent sample handling due to RNA stability issues. Similarly, the CRISPR-TB assay provides ultra-sensitive and rapid detection, even in low-bacterial load samples, within 1–2 h and can be adapted to identify drug-resistant strains (13, 24). However, both methods face challenges related to technical complexity, cost, and accessibility.

The TPAPy-Tre probe is incorporated into the mycobacterial cell wall by the Ag85 mycolyl transferase complex; detection of a fluorescent TPAPy-Tre signal in a whole mycobacterial cell therefore indicates a metabolically active organism (25). Prior studies of bacillary size, morphology, and staining characteristics have shown good agreement between patient samples and cultures of the laboratory strain, Mtb H37Rv (26). TPAPy-Tre offered the significant advantage of rapid detection within 2 h, which is faster than TB-MBLA and comparable to the amplification time of CRISPR-TB. TPAPy-Tre has a consistent correlation with bacillary load measured by CFU and TTP. Before treatment, when sputum bacillary load is high, the concentration of TPAPy-Tre-labeled fluorescence intensity is also high. As patients progress on treatment, both sputum bacillary load and TPAPy-Tre-labeled fluorescence intensity decline in a manner that mirrors each other. TPAPy-Tre could be used to monitor TB bacillary load during treatment with the advantage of providing results rapidly. As a fully quantitative assay for detecting viable Mtb, using 2 week TPAPy-Tre to reveal the actual bacterial load in samples would robustly monitor TB treatment response.

We found bacillary cell lengths in sputum to vary in a skewed distribution, with a median of 2.7 µm and an IQR of 2.4–3.0 µm before TB treatment, increasing to a median of 3.3 µm (IQR, 3.0–3.9) after TB treatment. This match reported cell-length distributions for bacilli in bioaerosols and blood in patients with pulmonary TB (15, 27, 28). In addition, we show a significant decrease in single bacilli fluorescence intensity and a significant increase in cell length over the observed period of tuberculosis treatment. The apparent elongation of *M. tuberculosis* bacilli in response to antimicrobial stress is tentatively suggestive of filamentation growth without division, which has been reported for other bacteria (15). And the apparent decrease in fluorescence intensity of *M. tuberculosis* bacilli in response to TB treatment is tentatively suggestive of a reduction in bacterial metabolic activity, which has been previously reported (14). At a quantitative level, imaging organisms from diagnosed TB patients before and after treatment initiation, as well as serially over the course of chemotherapy, might therefore offer a more rapid indication of drug efficacy, in effect affording the sensitivity results of sputum samples without the invasiveness of that procedure. Taken together, our results suggest that TPAPy-Tre microscopy is an informative and novel tool providing single-cell phenotypic information relevant to the pathobiology of severe MDR/RR-TB, in particular, the mycobacterial response to antimicrobial exposure *in vivo*, ascertain the impact of TB treatment on Mtb.

Limitations of this study include the single-center recruitment and small sample size in this study. As our clinical trial is still ongoing, the data generated in the short term will provide a more robust evaluation of the role of TPAPy-Tre microscopy in EBA studies. Because most patients remained sputum culture and TPaPy-Tre microscopy positive at the last observed time point, median time to sterilization of sputum by these assays could only be extrapolated; in the future, later time points should be assessed. Although the TPAPy-Tre microscopy method offers significant advances in characterizing Mtb and relative specificity for mycobacteria, it is labor-intensive and, as with all manual microscopy, requires subjective calls when classifying fluorescent objects as bacilli. An assay that can monitor TB treatment response more effectively than culture and provide results in less than 2 h has great potential to improve patient treatment management, particularly in those with drug-resistant tuberculosis. Future goals should include automation and high-throughput adaptations of the TPAPy-Tre microscopy.

In summary, TPAPy-Tre demonstrated comparable performance to both traditional solid and liquid culture in serially evaluating the vitality of bacteria, with the significant benefit of accelerated results in only a few hours. The unique capability of TPAPy-Tre microscopy to rapidly and visually assess the vitality of individual bacteria during tuberculosis treatment, along with its ability to deliver immediate feedback, might represent a substantial improvement over traditional methods. This new tool might apply to monitoring bacterial load throughout TB treatment, enabling early assessment of the bactericidal activity of anti-TB drugs. Further prospective studies utilizing TPAPy-Tre in a larger cohort, including individuals with drug-susceptible tuberculosis, are warranted to validate these findings and establish its role in monitoring treatment response in tuberculosis.

## SUPPLEMENTAL MATERIAL

Supplemental material spectrum.02457-24-s0001.docx Fig. S1 to S4; Table S1.

ASM does not own the copyrights to Supplemental Material that may be linked to, or accessed through, an article. The authors have granted ASM a non-exclusive, world-wide license to publish the Supplemental Material files. Please contact the corresponding author directly for reuse.
