# Supplementary material for: A cytoderm metabolic labeling TPAPy-Tre for real-time detection of vitality of Mycobacterium tuberculosis in sputum
Source: Microbiol Spectr. 2025 May 22;13(7):e02457-24. doi: 10.1128/spectrum.02457-24 (PMC12211060; doi:10.1128/spectrum.02457-24)
Supplement: Supplemental material — Fig. S1 to S4; Table S1. [file spectrum.02457-24-s0001.docx]

**Supplement**


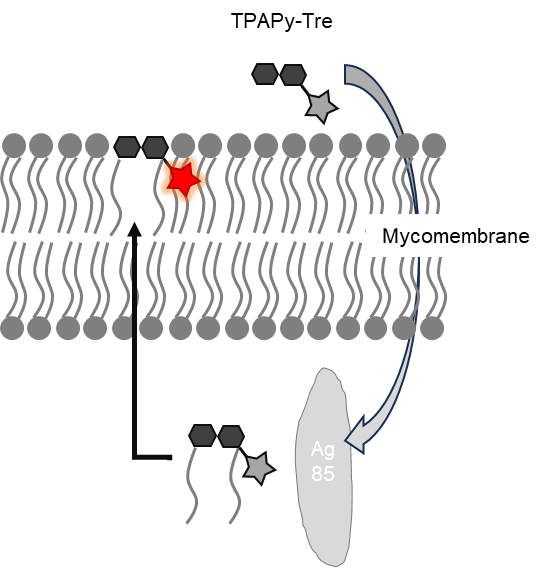


**Figure S1.** TPAPy-Tre integrated to the mycomembrane of Mycobacterium tuberculosis via the Ag85 complex, illuminates the bacterial cell wall brightly.

**Figure S2.** The receiver operating characteristic (ROC) curves generated using TPAPy-Tre-labeled fluorescence intensity were utilized to ascertain potential cut-off points for levels of positivity.

**Table S1:** Baseline Demographics and Clinical Characteristics of participants with MDR/RR-TB

|  | **Age (years)** | **Sex** | **Diabetes** | **NIH criteria signs and symptoms of tuberculosis > 2 ^a^** | **ESR (mm/h)** | **CRP (mg/L)** | **Xpert** | **AFB** | **TB-DNA** | **MGIT culture time to positive (h)** | **Solid culture** | **TPAPy-Tre-labeled FL intensity (a.u.)^b^** |
| --- | --- | --- | --- | --- | --- | --- | --- | --- | --- | --- | --- | --- |
| P1 | 47 | Male | Yes | Yes | 82 | 222.5 | Positive (Low) | + | Positive (CT 28.3) | 165.5 | Positive | Positive (102.6) |
| P2 | 40 | Male | No | No | ·· | 4.6 | Positive (Middle) | ++++ | ·· | 148.0 | Positive | Positive (292.9) |
| P3 | 76 | Male | No | Yes | 77 | 21.9 | Positive (Extremely low) | Scanty | Positive (CT 25.3) | 156.5 | Positive | Positive (233.0) |
| P4 | 35 | Male | No | Yes | 31 | 2.6 | Positive (Middle) | + | Positive (CT 23.5) | 136.0 | Positive | Positive (231.7) |
| P5 | 69 | Male | Yes | No | 89 | 82.5 | Positive (Middle) | Negative | Positive (CT 25.8) | ·· | Positive | Positive (246.4) |
| P6 | 62 | Male | No | Yes | 100 | 51.8 | Positive (High) | +++ | Positive (CT 16.2) | 167.0 | Positive | Positive (293.9) |
| P7 | 42 | Male | No | No | 50 | 22.5 | Positive (Middle) | ++ | Positive (CT 20.9) | 124.5 | Positive | Positive (459.5) |

All participants with multidrug-resistant/rifampicin-resistant tuberculosis, listed by participant ID. ^a^ Persistent cough, fever, night sweats, weight loss, and other respiratory symptoms. ^b^ Cut off value determined based on fluorescence values of none-tuberculosis population (Figure S2). NIH=National Institutes of Health. ESR=Erythrocyte sedimentation rate. CRP=C-reactive protein. Xpert=Xpert MTB/RIF. AFB=Acid-fast bacilli smear. IGRA=Interferon-gamma release assay. MGIT=Mycobacteria growth indicator tube. FL intensity= Fluorescence intensity. a.u. =Arbitrary units. ··=Missing data.


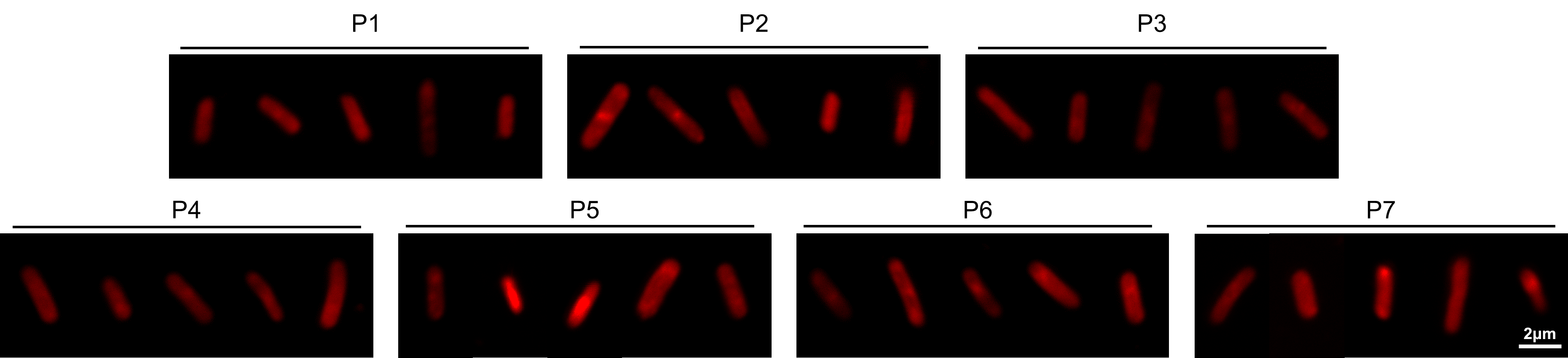


**Figure S3.** TPAPy-Tre metabolic labeling imaging of all patients using confocal laser scanning microscopy before TB treatment.


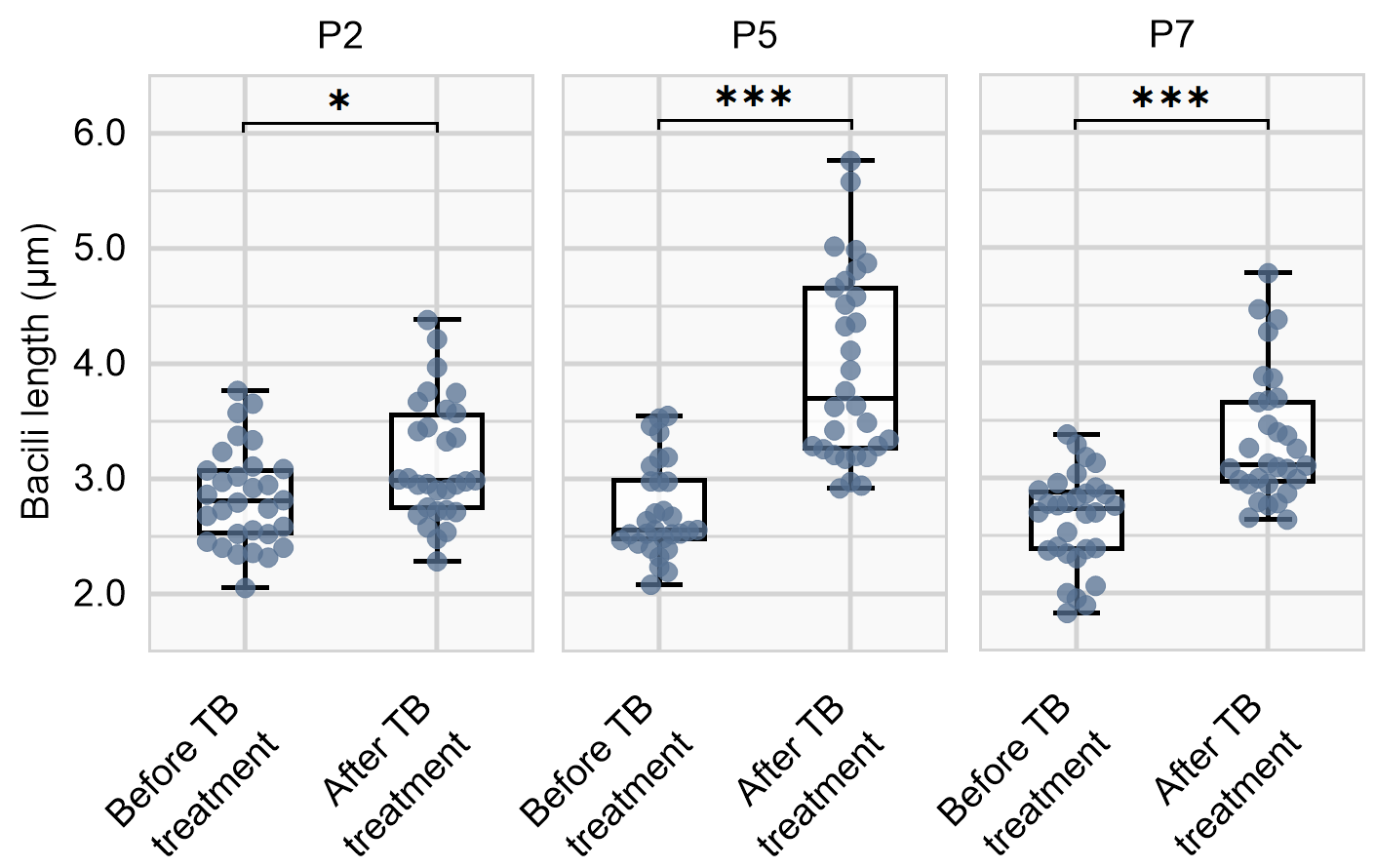


**Figure** **S4.** Confocal laser scanning microscopy analysis real-time changes in bacterial sugar metabolism labeling in sputum with TPAPy-Tre. Boxplots of bacterial cell-lengths at before and after treatment timepoints are presented.
